# Automatic text classification of drug-induced liver injury using document-term matrix and XGBoost

**DOI:** 10.3389/frai.2024.1401810

**Published:** 2024-06-03

**Authors:** Minjun Chen, Yue Wu, Byron Wingerd, Zhichao Liu, Joshua Xu, Shraddha Thakkar, Thomas J. Pedersen, Tom Donnelly, Nicholas Mann, Weida Tong, Russell D. Wolfinger, Wenjun Bao

**Affiliations:** ^1^Division of Bioinformatics and Biostatistics, National Center for Toxicological Research, U.S. Food and Drug Administration, Jefferson, AR, United States; ^2^JMP Statistical Discovery LLC, Cary, NC, United States; ^3^Boehringer Ingelheim Pharmaceuticals, Inc., Ridgefield, CT, United States; ^4^Department of Pharmaceutical Sciences, University of Arkansas for Medical Sciences, Little Rock, AR, United States; ^5^Department of Mathematics, The University of North Carolina at Chapel Hill, Chapel Hill, NC, United States

**Keywords:** anatomical therapeutic chemical classification (ATC), Matthews correlation coefficient (MCC), area under the curve, XGBoost, LightGBM, CatBoost, TF-IDF

## Abstract

**Introduction:**

Regulatory agencies generate a vast amount of textual data in the review process. For example, drug labeling serves as a valuable resource for regulatory agencies, such as U.S. Food and Drug Administration (FDA) and Europe Medical Agency (EMA), to communicate drug safety and effectiveness information to healthcare professionals and patients. Drug labeling also serves as a resource for pharmacovigilance and drug safety research. Automated text classification would significantly improve the analysis of drug labeling documents and conserve reviewer resources.

**Methods:**

We utilized artificial intelligence in this study to classify drug-induced liver injury (DILI)-related content from drug labeling documents based on FDA’s DILIrank dataset. We employed text mining and XGBoost models and utilized the Preferred Terms of Medical queries for adverse event standards to simplify the elimination of common words and phrases while retaining medical standard terms for FDA and EMA drug label datasets. Then, we constructed a document term matrix using weights computed by Term Frequency-Inverse Document Frequency (TF-IDF) for each included word/term/token.

**Results:**

The automatic text classification model exhibited robust performance in predicting DILI, achieving cross-validation AUC scores exceeding 0.90 for both drug labels from FDA and EMA and literature abstracts from the Critical Assessment of Massive Data Analysis (CAMDA).

**Discussion:**

Moreover, the text mining and XGBoost functions demonstrated in this study can be applied to other text processing and classification tasks.

## Introduction

Drug labeling documents are issued by regulatory agencies such as the United States Food and Drug Administration (FDA) and Europe Medical Agency (EMA) to communicate safety and efficacy information for approved drugs available to the public. Comprehensive information such as indications, contraindications, and warnings for adverse drug reactions (ADRs) is included in drug labeling as a reference for healthcare professionals and patients (Watson and Barash, 2009; McMahon and Preskorn, 2014). The FDA and EMA have approved over 130,000 labeling documents by 2022, creating a vast repository of regulatory text data for regulatory agency reviewers and scientific researchers (Hoffman et al., 2016; Fang et al., 2020; Wu et al., 2021).

Drug-induced liver injury (DILI) is a common adverse drug reaction documented in drug labeling and has been recognized for its significant role in drug failure and withdrawal. Chen et al. (2011, 2016) and Wu et al. (2022) used text data from FDA drug labeling to annotate DILI risk in humans for 1,036 drugs. They manually searched the data using a set of predefined keywords and followed with manual curation to generate their annotations. Although manual curation ensures the specificity and usefulness of the information, it requires significant time and effort, including reading, understanding, and classifying the information for each drug, and is subject to the individual judgment and expertise of the reviewer. Drug labeling documents are updated regularly based on new findings from pharmacovigilance studies and case reports in literature (Food and Drug Administration, 2015a,b). Given the large volume of labeling documents, it is highly challenging to routinely reassess and update safety information manually. An automated, simplified text classification approach for processing text data in labeling and other documents would streamline the process and conserve reviewer resources.

Text mining is a valuable approach for gathering ADR information in drug labeling for comparative analysis during drug evaluation or scientific research (Fang et al., 2020). To this end, the standard ADR terms from the Medical Dictionary for Regulatory Activities (MedDRA) and Systematized Nomenclature of Medicine (SNOMED) are commonly used to create a document-term matrix (DTM), which captures the frequency of terms in documents (Wu et al., 2019; Demner-Fushman et al., 2021; Wu et al., 2022). The DTM can then be used as input for machine learning algorithms to classify drug labeling documents based on ADR risk and identify important features that contribute to the classification.

Tree ensemble models such as XGBoost (Extreme Gradient Boosting) (Chen and Guestrin, 2016), LightGBM (light gradient-boosting machine) (Ke et al., 2017) and CatBoost (Categorical Boosting) (Prokhorenkova et al., 2018) are machine learning algorithms commonly used for classification and regression (Shwartz-Ziv and Armon, 2022). XGBoost is a decision tree-based method that utilizes the principle of ensemble learning to make predictions. This process combines decisions from multiple models to produce a final prediction, in which the results of one model serve as input for the next, allowing the new model to correct errors made by the previous ones. XGBoost is widely used in a broad range of fields given its advantages in flexibility and interpretability (Zhang et al., 2018; Tahmassebi et al., 2019; Li et al., 2020; Pandi et al., 2020; Chatterjee et al., 2021).

In this study, natural language processing was employed to create a DTM. The matrix was constructed using MedDRA or FDA Medical Query (FMQ) preferred terms (PTs) retrieved from the drug labeling documents and scientific abstracts. The XGBoost algorithm was then utilized to predict DILI from drug labeling documents and abstracts based on the DTM. The prediction model was evaluated through cross-validation, and the significance of standard terms was ranked and assessed for their relevance to DILI. The efficacy of this automatic text classification approach was verified through testing on FDA drug label data, EMA drug label data, and scientific literature data retrieved from the Critical Assessment of Massive Data Analysis (CAMDA). This approach was finally confirmed using a model generated by the FDA drug label dataset to predict DILI potential in the EMA drug label dataset.

## Materials and methods

### Datasets

The first dataset was generated from the FDA drug labeling documents of the DILI-rank dataset (Chen et al., 2016). The drug labeling documents were retrieved from the FDALabel database^1^ (Fang et al., 2020) with chemical structures. The drug labels were annotated for their DILI potential manually (Chen et al., 2011). Text of “Warnings and Precautions” sections of drug labeling documents were extracted for this study. After removing those without labeling (e.g., withdrawn drugs) or chemical structures (e.g., biological products, mixtures), 678 unique prescription drugs approved by the FDA were identified. This data set contained 238 (35%) drug labeling documents that have DILI potential (defined as 1) and 440 (65%) that have no DILI potential (defined as 0). This dataset is hereafter referred to as the “FDA dataset.”

The second dataset was drug labeling documents of 277 unique prescription drugs from EMA that were retrieved from Electronic Medicines Compendium^2^ (Annex, 1999). The drug labels were annotated for their DILI potential manually as for FDA drug labels (Chen et al., 2011). Text of “Warnings and Precautions” sections of EMA drug labeling documents were extracted for this study. This data set contained 132 (48%) drug labeling documents that have DILI potential (defined as 1) and 145 (52%) that have no DILI potential (defined as 0). This dataset is hereafter referred to as the “EMA dataset.”

The third dataset was retrieved from the CAMDA. This data set was originally published for the “Literature AI for Drug Induced Liver Injury” challenge in 2021.^3^ This data set contained 12,187 abstracts of published papers from PubMed.^4^ Each of the abstracts were annotated for DILI association by the experts in the NIH LiverTox. This data set contained 5,161 (42%) abstracts that were associated with drugs with DILI potential (defined as 1) and 7,026 (58%) abstracts that were associated with drugs without DILI potential (defined as 0). This dataset is hereafter referred to as the “CAMDA dataset.”

### Software package used

Natural language processing used Text Explorer (TE) function, and model comparisons were conducted via predictive model screening platform in the JMP Pro Statistical Discovery software package (JMP Pro v17, https://www.jmp.com/) in this study. The XGBoost add-in tool can be downloaded from https://community.jmp.com/t5/JMP-Add-Ins/XGBoost-Add-In-for-JMP-Pro/ta-p/319383 and installed into the software.

### Construction of document term matrix (DTM) by natural language processing

**Figure 1:**
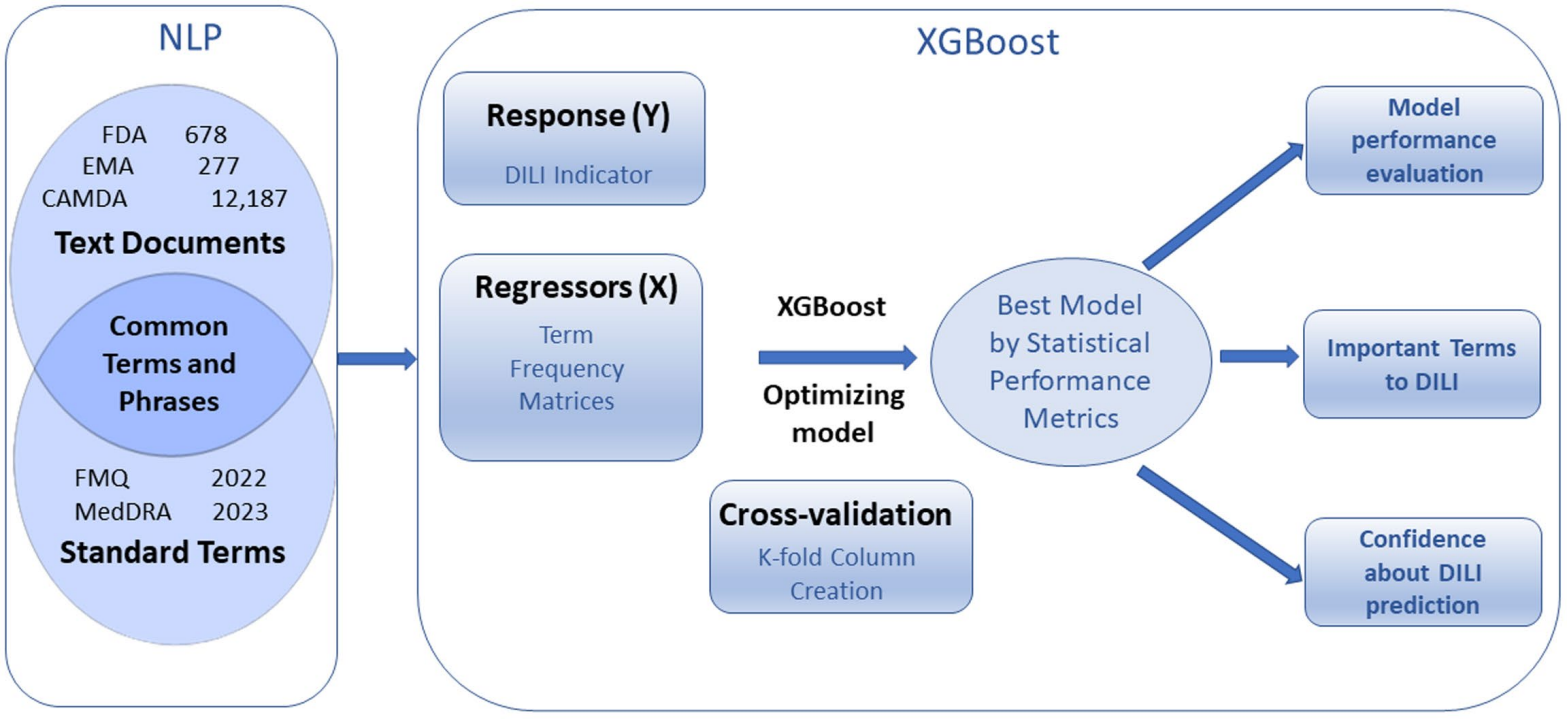
Overview of text document analysis procedure. Natural Language Processing (NPL) generates term frequency matrices that are used to predict DILI indicator with cross validation. Optimized XGBoost models produce statistical performance metrics, important terms to DILI, and confidences about prediction.

The FDA, EMA and CAMDA datasets were converted to a DTM using TE separately, the natural language processing function in the software the natural language processing here consisted of the curation of standardized terms, tokenization, and generation of a DTM. (Figure 1).

### Curation of standardized terms

The medical-related terms were curated by the MedDRA or FMQ (Andrade et al., 2019; FDA, 2022) PT list. The terms and phrases in FDA, EMA or CAMDA that matched standardized terms were extracted from documents for further analysis. This is achieved by setting all the terms and phrases identified by TE as stop words first, and then adding back terms and phrases and stop word local exception that match MedDRA PT.

### Tokenization

Some related terms were combined into a single term by tokenization. For example, allergic hepatitis, autoimmune hepatitis, chronic hepatitis, chronic hepatitis b, chronic hepatitis c, hepatitis b surface antigen, and hepatitis c were all recoded to hepatitis; acute hepatic failure was recoded to hepatic failure. Some terms were processed by stemming; for example, aminotransferases, transaminases, liver enzymes and liver function tests were recoded to aminotransferase, transaminase, liver enzyme and liver function test, respectively.

### Term frequency-inverse document frequency (TF-IDF)

The DTM was constructed using weights computed by Term Frequency-Inverse Document Frequency (TF-IDF) for each included word/term/token. Specifically, this weighted TF-IDF is calculated by the *log* value of the frequency of a standardized term vs. the total number of documents in the corpus. This algorithm reduced the weights of the highly frequent terms and added weight to less frequent terms in each document.

### Classification modeling by XGBoost

XGBoost decision tree machine learning library uses DTM of standardized terms to predict DILI classification. The XGBoost modeling process with cross validation includes cross validation *k*-fold column creation, XGBoost model setup, and modeling optimization including adjusting iteration number and applying autotune with advanced options (Supplementary Figure S1).

### XGBoost model setup

The model was set up using DILI indicator as the Y response, DTM as the X regressor and defining the number of cross-validation folds and number of folds within each column for validation. Here, we used minimal term frequency as 1 for FDA and EMA dataset and as 10 for CAMDA data for generating DTM showed in Supplementary Figure S1 to predict DILI indicator as Y responses and run 10 times of 5-fold validation.

### XGBoost model optimization

The different iterations were performed according to the iteration history for the default condition. The optimization of the model can also be achieved by using autotune with advanced options (Supplementary Figure S1).

### Cross validation

To reduce the risk of overfitting, a cross validation approach was used to evaluate model performance. The data set was divided into 5 roughly equal folds, and each level as a hold-out set. A group of 10 sets of 5 folds were prepared such that each fold was geometrically orthogonal to each fold in the other sets. The folds were stratified by the DILI indicator variable, which assures that approximately the same proportion of DILI occur in each subset.

### Model autotune with advanced options

The XGBoost model has a set of built-in hyperparameters that come with default values and suggested ranges for autotune with basic and advanced options (Supplementary Figure S1). The value and range of the hyperparameters can be modified.

Autotune offers a list of hyperparameters with default values and range, including max_depth for 3 (1–7), subsample for 1 (0.5–1), colsample by tree for 1 (0.5–1), min child weight for 1 (1–3), alpha for 0 (0–0.5), lambda for 1 (0–2), learning rate for 0.1 (0.05–0.2), and iterations for 30 (20–50) with number of design points as 10 and number of inner folds as 2. The more advanced options are also available for change. Those hyperparameters can be modified to control or prevent over-fitting and make models reasonably conservative, as overfitting is the most common issue in the machine learning analysis (Tahmassebi et al., 2019; Li et al., 2020; Pandi et al., 2020; Chatterjee et al., 2021).

### Statistical performance metrics

Six statistical metrics were used to assess XGBoost performance; these are Accuracy (ACC), area under the receiver operating characteristic curve (AUC), Matthews correlation coefficient (MCC), sensitivity, specificity and precision for training and validation sets for DILI indicator as binary Y response.


$$ACC=\left(TP+TN\right)/\left(TP+TN+FP+FN\right)$$

$$MCC=\frac{\left(TP*TN-FP*FN\right)}{\sqrt{\left(TP+FP\right)*\left(TP+FN\right)*\left(TN+FP\right)*\left(TN+FN\right)}}$$

$$\text{Sensitivity}=TP/\left(TP+FN\right)$$

$$\text{Specificity}=TN/\left(TN+FP\right)$$

$$\text{Precision}=TP/\left(TP+FP\right)$$

Here, TP = True Positives; FN = False Negatives; TN = True Negatives; FP = False Positives.

AUC is the area under the ROC (Receiver Operating Characteristic) curve. The ROC curve plots sensitivity (true positive rate) on the Y axis vs. specificity (true negative rate) on the X axis. Sensitivity shows how well the model detects positives, that is, the ratio of true positives to true positives plus false negatives. Specificity defines how well the model avoids false alarms, which is ratio of true negatives to true negatives plus false positives. The ROC curve is constructed by plotting sensitivity versus specificity over a range of cutoff values applied to predicted probabilities. AUC can be interpreted as a measure of sorting efficiency. An AUC of 1.0 indicates perfect sorting and a value of 0.5 indicates no predictive performance.

### Predicting new documents

The prediction formula of the selected model for existing documents can be saved and applied to new documents to generate prediction probability for DILI potential associated with drugs. First, the document term frequency (DTF) for new documents can be created with the same text mining process as described above. Second, concatenate new documents with the DTF to the existing documents. Third, apply the saved prediction formula to the new documents. The prediction probability for DILI potential associated with the drug for each new document will be generated.

### Comparing XGBoost with other predictive models

Predictive model screening was employed to compare multiple predictive models. The nested cross validation was used with k as 10 and L as 5 to match the validation in XGBoost.

## Results

### Data preprocessing

**Figure 2:**
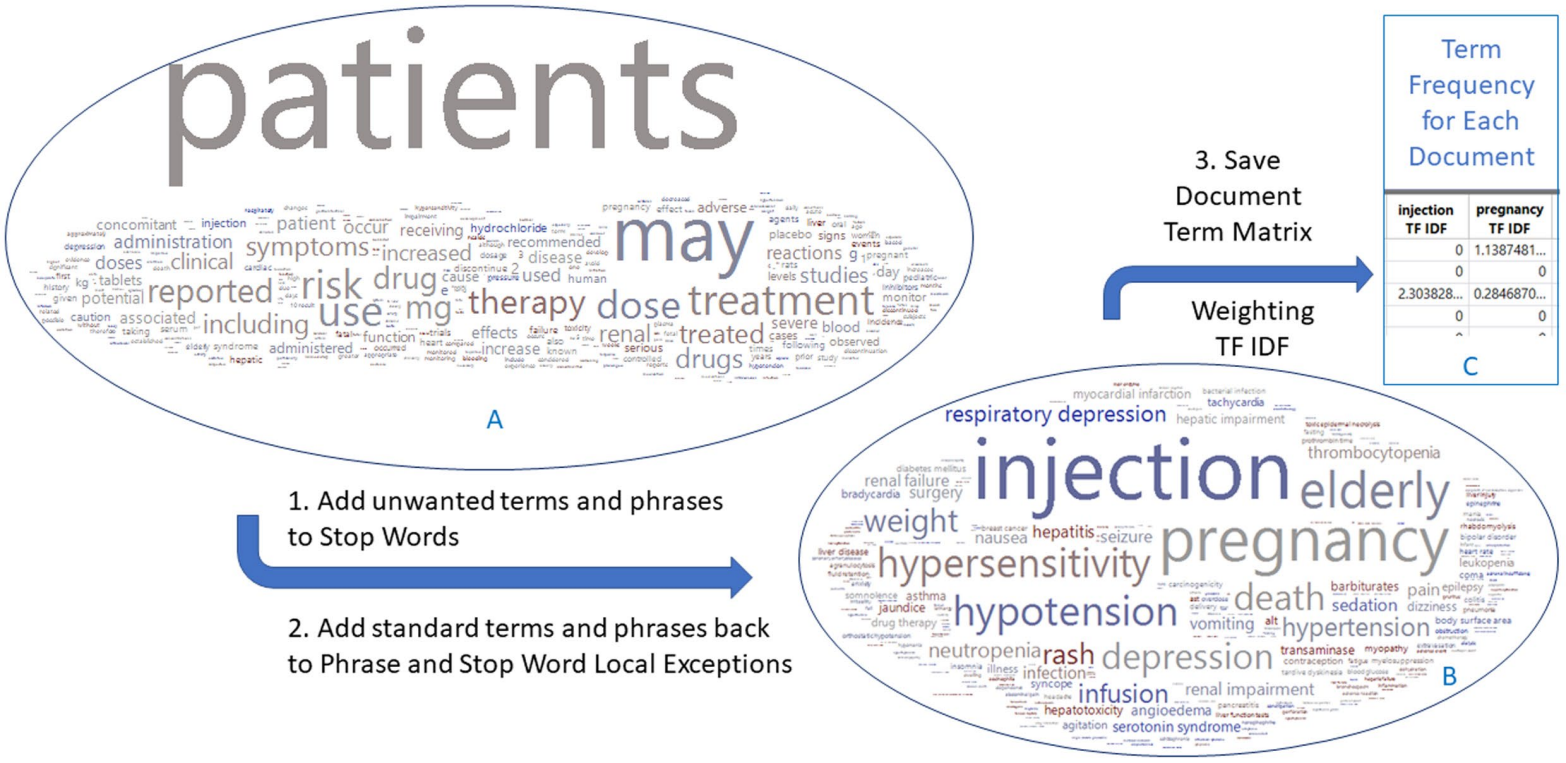
Flowchart to generate a document term matrix from FDA drug label documents. The 678 drug labels have 14,600 terms initially, which are reduced to 1,443 terms that match the MedDRA preferred terms. Word clouds are colored by DILI indicator: red for 1 and blue for 0. The EMA and CAMDA data used the same approach.

The text sections of “warning and precaution” in FDA drug labeling documents for 678 drugs were imported into the TE platform, yielding 14,600 terms and phrases out of 25,916 unique PTs in MedDRA 26.0, with the most frequent terms being common words such as ‘patients’, ‘may’, ‘treatment’ (Figure 2). These common words were not medically related and should be removed. Instead of utilizing the common language processing techniques such as tokenizing, phrasing, terming, and stemming or manually defining the terms and phrases, we took advantage of the known MedDRA standard terms to narrow to medically related terms in three steps (Figure 2). First, all the terms and phrases (14,600) were removed by adding them as stop words in Text Explorer. Second, terms and phrases were matched with MedDRA PTs by using the software’s Manage Phrase and Manage Stop Word functions, resulting in 1443 medical related terms and phrases of standard terms. Finally, each term and phrase were assigned values for each drug document, and a document term matrix weighted with TF-IDF was generated for the use in XGBoost modeling. The same process was applied to EMA and CAMDA datasets.

### XGBoost modeling for DILI classification

The parameters for XGBoost modeling were optimized using cross-validation. We explored the effects of term frequency and validation first for the FDA dataset. Using a term frequency of 1, 2 or 30 for drug labeling data yielded no significantly different results. The 10-fold validation resulted in a significantly higher AUC with a smaller variation compared to either 3 or 5 validation folds as measured by ANOVA (data not shown). Therefore, we used a term frequency of 1 (all the terms) for FDA and EMA datasets and a term frequency of 10 for CAMDA dataset to save the model calculation time. The 10-fold validation method for optimization conditions was run for all three datasets. The results include statistical performance metrics, a ranking of importance of terms, and a prediction formula for new data.

**Figure 3:**
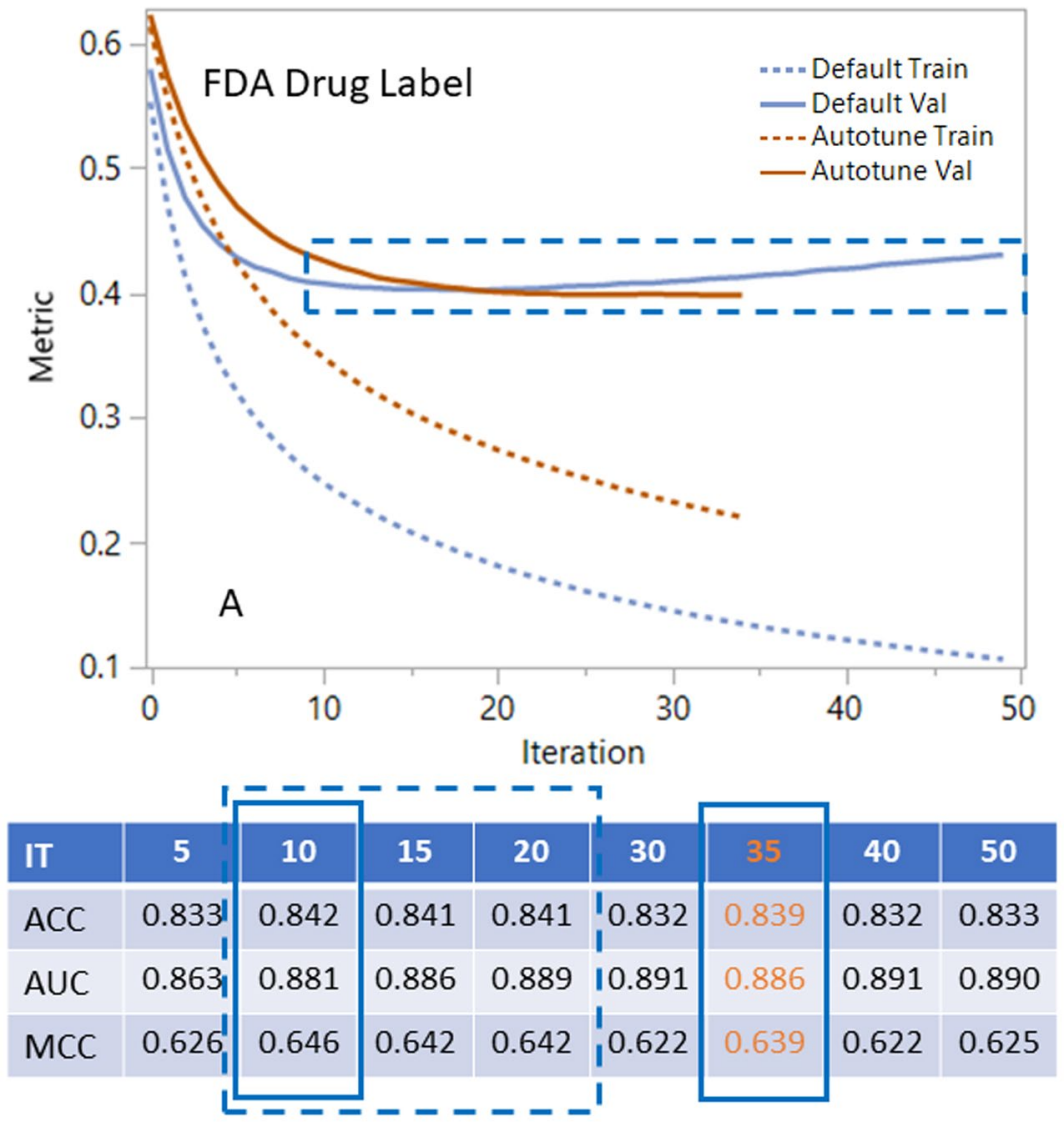
Using iteration history curves to optimize XGBoost performance for FDA drug label dataset. The best models lie in the dashed rectangular box of ranges of the validation curves for default parameters (solid blue) or autotune (solid brown). The dashed lines are for the corresponding training curves.

The XGBoost generates an iteration history that can assist researchers in choosing the proper number of iterations. For the FDA dataset shown in the plot and table in Figure 3, the default condition (Supplementary Figure S1C) with different iterations shows that validation curves (solid blue line) rapidly decline and then level off between 10 to 20 (Figure 3). Statistical metrics such as ACC, AUC and MCC for validation are very similar between 10 to 20 iterations with the peak statistical metrics at iteration 10. After 20 iterations, the validation curve started to rise, and the statistical metrics declined. When we applied autotune with default options (Supplementary Figure S1D), the validation curve (solid yellow line) leveled off with a different slope. The autotune generates the optimized condition (Figure 3) with iteration at 35 and almost the same statistical metrics as the default peak condition at iteration 10. The EMA dataset had very similar iteration history curve with peak range in 10–20. The autotunes for FDA and EMA datasets have similar iterations at35 and 36. The CAMDA dataset needed 300 iterations to reach the optimized condition with the default condition and 638 iterations for autotune (Supplementary Figure S2).

### Model performance evaluation

The statistical metrics such as ACC, AUC, MCC and root mean square error (RMSE) are listed in the Table 1 for training and validation of three datasets. Three other core concepts that are often used to evaluate the accuracy of models include sensitivity, specificity, and precision from different angles, which are also listed in Table 1. The results in Table 1 were generated by 10 iterations for the FDA and EMA datasets and 300 iterations for CAMDA that are optimized default condition, plus the results were generated by autotune. The differences of the statistical metrics between defaults and autotunes for each dataset are ranged from 0 to 0.036, mostly less than 0.01. The differences of the three core concepts between defaults and autotunes for each dataset are ranged from 0.002 to 0.099, mostly less than 0.05.

**Table 1:** Statistical metrics include ACC, AUC, MCC, RMSE, Precision, Sensitivity and Specificity for FDA, EMA and CAMDA datasets.

Dataset		Parameters	ACC	AUC	MCC	RMSE	Precision	Sensitivity	Specificity
FDA Drug Label	Training	Default	0.913	0.977	0.812	0.256	0.979	0.769	0.991
		Autotune	0.931	0.983	0.848	0.236	0.970	0.828	0.986
	Validation	Default	0.842	0.881	0.646	0.348	0.862	0.656	0.943
		Autotune	0.839	0.886	0.639	0.344	0.856	0.651	0.941
EMA Drug Label	Training	Default	0.971	0.996	0.942	0.177	0.971	0.955	0.986
		Autotune	0.975	0.997	0.950	0.165	0.934	0.8560	0.945
	Validation	Default	0.903	0.958	0.807	0.280	0.985	0.962	0.986
		Autotune	0.903	0.957	0.805	0.281	0.920	0.871	0.931
CAMDA Abstract	Training	Default	0.883	0.946	0.762	0.298	0.931	0.780	0.958
		Autotune	0.864	0.927	0.722	0.338	0.905	0.757	0.942
	Validation	Default	0.844	0.904	0.680	0.319	0.881	0.729	0.928
		Autotune	0.837	0.897	0.666	0.344	0.871	0.722	0.921

The drug label data has default parameters with 10 iterations and autotuned parameters with 35 iterations. The CAMDA data has default parameters with 300 iterations at 300 and autotuned parameters with 638 iterations.

### Ranking of term importance

The relative importance of the standardized terms is ranked by three related measurements: splits, gain, and cover. Splits represents the number of times that variable is used to split a branch in a tree. Gain is the average improvement in objective function for splits involving that variable, and Cover is the amount of data covered by splits involving that variable. Here, the gain is used to rank the importance of the selected terms as shown in Table 2. The top five terms of MedDRA are liver damage related keywords classified in Chen et al. (2016), including hepatotoxicity (#1), hepatitis (#2), hepatic failure (#3), liver injury (#4) and jaundice (#5). Another group are immune-related terms, such as toxic epidermal necrolysis (#7), thrombocytopenia (#8), and eosinophilia (#9). Blood urea (#6) and oliguria (#10) that could be related to kidney, are also in the top 10 list.

**Table 2:** Summary and list of top 10 features by gain for FDA drug label data according to MedDRA 26 and FMQ 2.1.

Feature	MedDRA26 Gain Rank	FMQ 2.1 Gain Rank	Terms
Hepatotoxicity	1	1	DILI Keyword
Hepatitis	2	2	DILI Keyword
Hepatic failure	3	3	DILI Keyword
Liver injury	4	4	DILI Keyword
Jaundice	5	5	DILI Keyword
Blood urea	6		Renal Dysfunction
Bone marrow depression		6	Immune System
Toxic epidermal necrolysis	7	8	Immune System
Thrombocytopenia	8		Immune System
Carcinoma		9	Cancer
Eosinophilia	9	10	Immune System
Oliguria	10	7	Renal Dysfunction

The top five features are DILI keywords. Other five features are related to immune system or renal dysfunction.

We also used FMQ to replace MedDRA as the standard terminology for the construction of the DTM and performed XGBoost with default condition at 10 iterations. FMQ was recently released by the FDA to standardize MedDRA PT groups according to medical concepts such as combining “initial insomnia,” “middle insomnia,” “early morning awakening,” to “insomnia” (FDA, 2022; FDA, 2023). The FMQ focuses on safety signal detection in clinical trial datasets. The 10 most important FMQ terms are also listed in Table 2. The top 5 terms were the DILI keywords with the same ranking as with MedDRA. The bone marrow depression (#6) and carcinoma (#9) are different from MedDRA.

**Figure 4:**
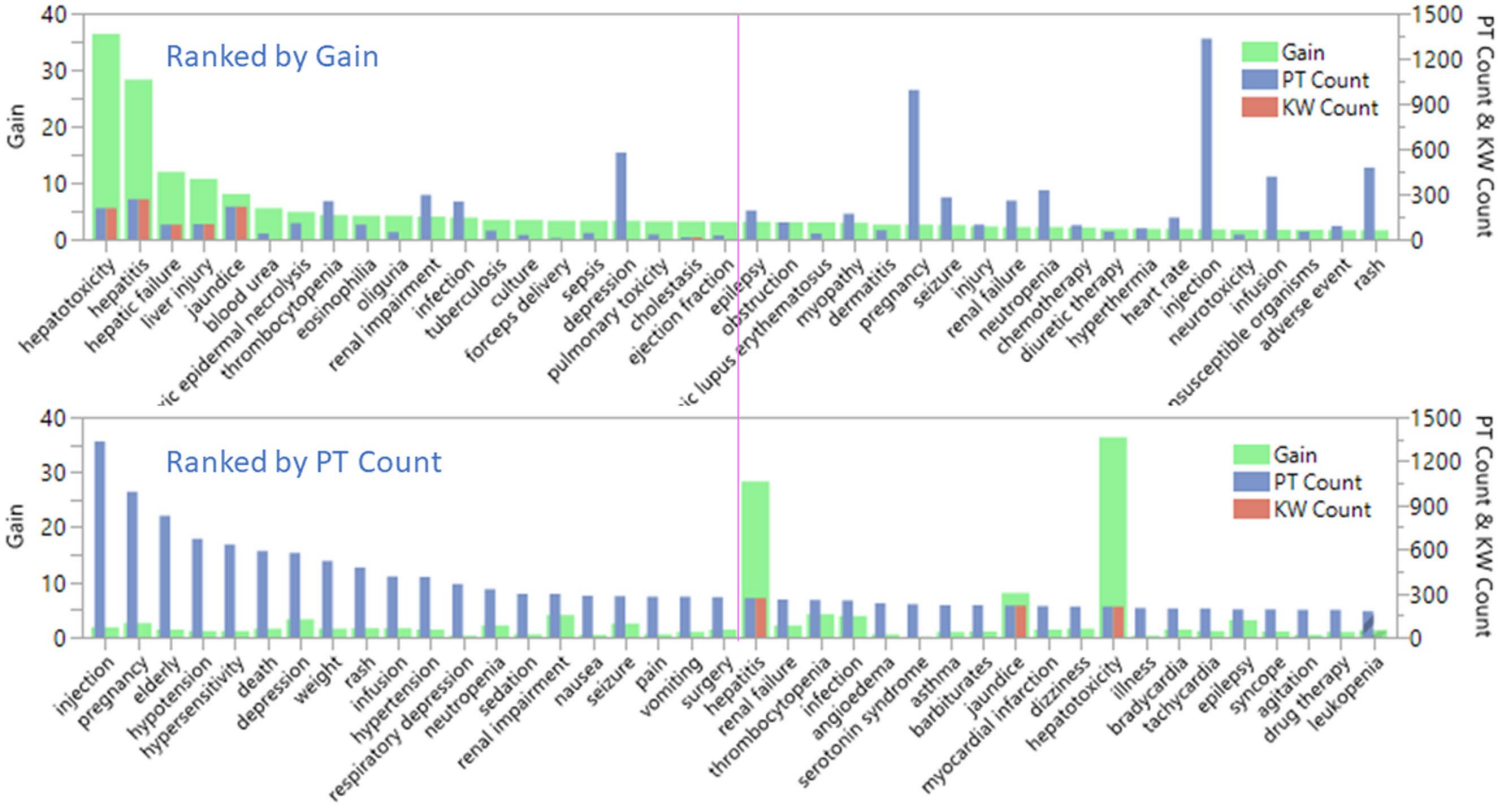
Comparison of the selected preferred terms by term count and term importance, as measured by gain for FDA drug label dataset.

The bar charts in Figure 4 showed the relationship between term count and term importance, as measured by gain. The value of gain is on the left Y axis and PT count and DILI keyword (KW) count are on the right Y axis. Only nine out of 58 KWs matched MedDRA PTs. The top plot was sorted by top 40 gain and bottom plot was sorted by top 40 PTs counts. The pink line is used to indicate top 20 terms. The top plot showed that first five plus cholestasis at top 19 are KWs. The bottom plot showed that the top 20 PT were not related to DILI. The top 10 PTs with count between 1,334 to 416 is 5 to 1.5 times greater than the term count of hepatitis (268). Hepatitis has the highest count in the KWs but is ranked #21 in all the terms. That means the importance of terms selected by the XGBoost modeling was not solely based on term count.

### Prediction of DILI for the EMA dataset

The prediction of the DILI indicator in the EMA dataset was based on the presence of terms and phrases that matched the MedDRA PT, where a term frequency of 1 was defined. XGBoost was used with the default conditions, varying the number of iterations, or using autotune (refer to Supplementary Table S1 for details). The validation curve for the iteration history of the EMA data resembled that of the FDA dataset, leveling off within the range of 10 to 20 iterations (data not shown). The statistical metrics for iterations 10, 15, and 20 were all within a negligible difference of 0.005, similar to those seen for the FDA dataset. Refer to Table 1 for a comprehensive overview of the statistical metrics specifically for the EMA data at iteration 10 under default conditions.

### Prediction of DILI for the CAMDA dataset

The data set from CAMDA was processed to construct DTM and XGBoost modeling similar to that done for the previous drug label data.

The results from the CAMDA dataset showed a similar pattern to the drug label dataset (Supplementary Figure S2), with DILI relevant terms dominating the top 10 important terms selected by XGBoost gain rank. The processing of the CAMDA dataset took 16 h to complete and the cross-validation results are shown in Table 1. An ACC of 0.844 or 0.837, AUC of 0.904 or 0.897, and MCC of 0.680 or 0.666 for default condition with iteration at 300 or autotune with iteration at 638. Additionally, immune-related terms such as rash, and rheumatoid arthritis, were also found among the top 10 important terms. These results indicate that the XGBoost modeling was successful in identifying relevant terms for DILI prediction using the CAMDA dataset as well.

### Comparison of model predictions for the FDA and EMA datasets

**Figure 5:**
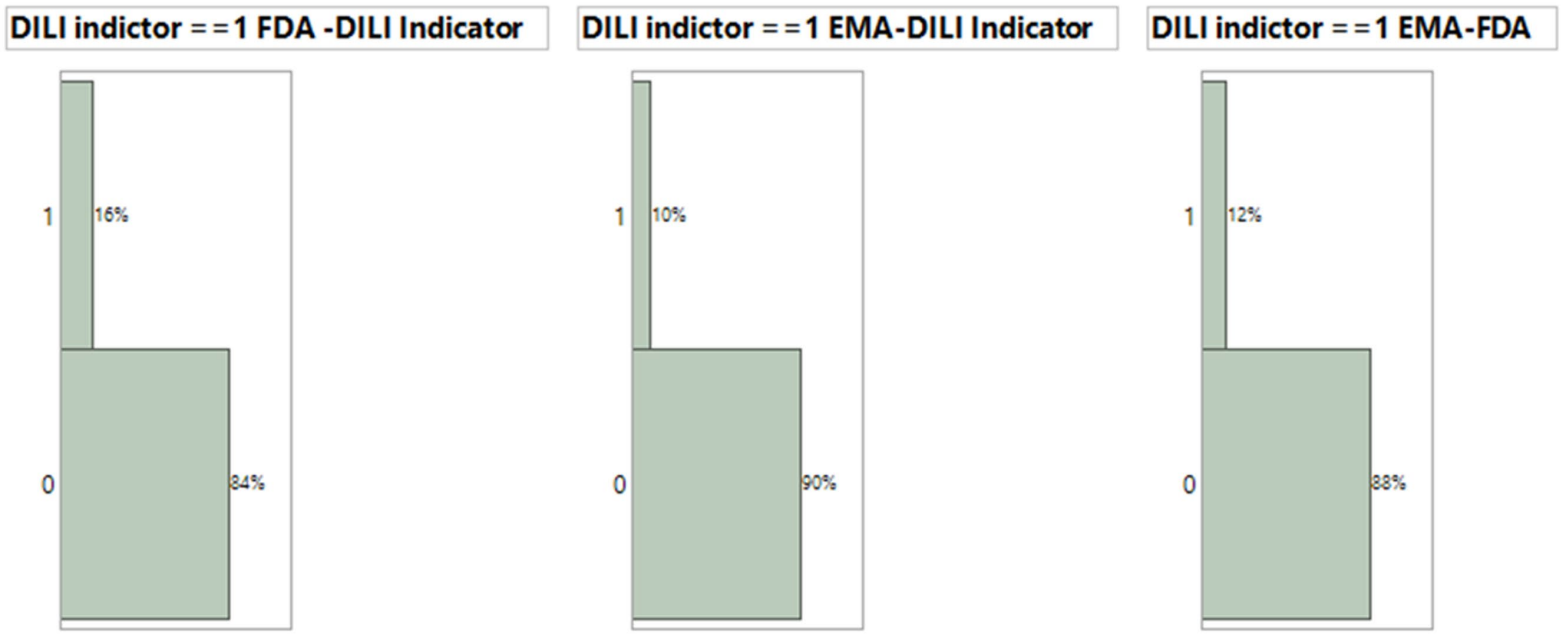
Comparison of consistency for XGBoost model results using default condition with 10 iteration and DILI indicator. The consistency between model prediction by EMA drug label or FDA drug label and DILI indicator and between EMA and FDA prediction.

Both the FDA dataset and EMA dataset serve as drug label datasets. Among the 1,443 terms and phrases from the FDA dataset and the 1,190 terms and phrases from the EMA dataset, there were 821 common terms and phrases that matched MedDRA PTs. We employed the FDA dataset model condition, using XGBoost with default parameters and 10 iterations, along with these terms and phrases, to generate prediction probabilities for each document from both FDA and EMA datasets. The prediction probabilities were rounded up to either 1 or 0. We evaluated their consistency by subtracting the rounded-up prediction probabilities from the DILI indicator, which has been defined by experts to assess the agreement between the model results and DILI indicators for each drug. A subtraction result with an absolute value of 0 indicates that both the model and indicator evaluate the drug’s DILI potential similarly. On the other hand, a subtraction result with an absolute value of 1 suggests a different classification between the model and indicator regarding the drug’s DILI potential. In the FDA dataset model, 16% or 45 out of 277 drugs were inconsistently classified (Figure 5, Left). Additionally, when comparing the model results for EMA dataset drugs with the EMA terms and phrases, using XGBoost default conditions and iterations, against the DILI indicators, we found that 10% or 27 out of 277 EMA drugs were inconsistently estimated (Figure 5, Middle). Lastly, there was a 12% inconsistency observed between the 1,190 EMA terms and phrases used to predict EMA data and the 821 common terms and phrases shared between the FDA and EMA datasets (Figure 5, Right).

### Consistency between experts’ annotation and XGBoost classification

**Figure 6:**
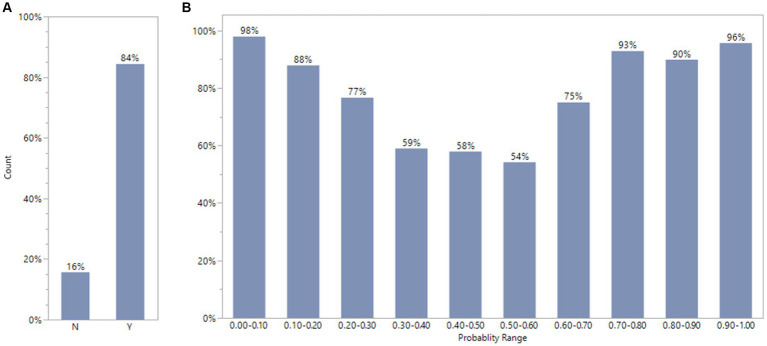
Comparison of consistency between experts’ opinions and XGBoost model classification for FDA drug labels using default condition with 10 iterations. The plot A showed that FDA drug labels has 84%(Y) consistency between XGBoost model results and experts ‘estimation. The plot B displays the percentage of consistency in different probability range by XGBoost model.

We compared consistency between expert opinion (DILI Indicator) and XGBoost model classification for FDA drug labeling using default condition with 10 iterations. The consistency evaluation method was described above. The 572 out of 678 or 84% FDA drugs label dataset were consistent between expert classification (DILI Indicator) and XGBoost model result (Figure 6A). To investigate further, we divided the model output probability into 10 ranges between 0 and 1. The lowest range, 0.00 to 0.10 means XGBoost model classifies the possibility to be DILI potential is very low. The highest range, 0.90 to 1.00, means XGBoost model classifies the possibility to be DILI potential is very high. Figure 6B indicates that for the probability ranges from 0.00 to 0.20 and from 0.70 to 1.00, the consistency rates were above or close to 90%; for probability ranges from 0.30 to 0.60, the consistency rates were about 55%; and for probability ranges from 0.20 to 0.30 and from 0.60 to 0.70, the consistency rates were about 75%.

### Comparison of XGBoost and other predictive models

We employed a predictive model screening platform to compare multiple predictive models. The nested cross validation was used with k as 10 and L as 5 to match the validation in XGBoost. The validated AUG results are shown in Table 3 The validated AUC for XGBoost, boosted tree, bootstrap, decision tree, neural boosted and support vector machines (SVMs) comparison are 0.881, 0.873, 0.872, 0.780, 0.785 and 0.764, respectively.

**Table 3:** Predictive model comparison for FDA drug label dataset.

	XGBoost	Boosted Tree	Bootstrap Forest	Decision Tree	Neural Boosted	SVM
Validation AUC	0.881	0.873	0.872	0.780	0.785	0.764

The validated AUC for XGBoost and five predictive models, includes boosted tree, bootstrap, decision tree, neural boosted and support vector machines (SVMs), from the predictive model screening platform were compared.

### Prediction errors analysis

Some drugs are found to have inconsistent DILI classifications between the model and expert reviewers. For example, Epirubicin was defined as DILI indicator of 1 by expert reviewers but predicted as 0 by the model with a high confidence (0.867). The text explorer using the selected KWs was run for this drug. Only two keywords, aspartate aminotransferase (AST) and hepatic impairment, showed up twice for Epirubicin in the word cloud (Supplementary Figure S3 left). Upon reviewing the sentences including those keywords (Supplementary Figure S3 right), it was found the sentences suggest precondition for hepatic impairment, not drug-induced results. Notably, human experts use the information from the multiple sections in drug label to determine DILI indicators, while our study only used the “warning and precaution” section in the drug label. This might be another reason for the inconsistencies between the model prediction and DILI indictors.

## Discussion and conclusions

In this study, we utilize artificial intelligence tools to classify whether contents from drug labeling documents and scientific abstracts are DILI related. We combined text mining and XGBoost models, taking advantage of standard PTs to simplify the elimination of the common/stop words. XGBoost models demonstrate excellent performance in classifying DILI, achieving AUC scores above 0.88 in cross-validation for both drug labels and the CAMDA datasets. Our results show that the DILI-related terms are the most critical contributors to DILI risk classification. Term frequency is not a significant factor, as feature importance did not correlate with term frequency. An important feature of the modeling workflow was to substitute PTs of MedDRA frequency to generate the DTM, suggesting that the results were not dependent on a specific set of standardized terms or corpus terms. Adding chemical descriptors as predictors did not significantly improve model performance (results not shown). Therefore, DILI-related standard terms appear to be the key factors for classifying whether the input text documents are relevant to DILI.

The top 5 important terms (MedDRA or FMQ) to contribute to XGBoost prediction are pre-selected keywords for manually annotating DILI by reviewers (Chen et al., 2011). Other top ranked terms, such as toxic epidermal necrolysis, thrombocytopenia, eosinophilia, depression are related to certain DILI mechanisms, as DILI can be linked to immune-mediated ADRs, such as drug reaction with eosinophilia and systemic symptoms, or cutaneous ADRs, including Stevens-Johnson syndrome and toxic epidermal necrolysis (Andrade et al., 2019). This suggests that the automatic text classification model can capture the underlying mechanistic relationship between DILI and immune disorders/skin reactions. Furthermore, renal dysfunction is commonly associated with liver diseases, especially in case of direct involvement in multiorgan acute illness or secondary to advanced liver disease (Betrosian et al., 2007). The model identifies several terms related to renal damage, such as blood urea and oliguria, as having high importance in model prediction, providing valuable mechanistic information for further investigation.

In terms of the document-term matrix, the FDA dataset uses 1,443 features and EMA used 1,190 features with a term frequency of 1, while the CAMDA dataset uses 740 features with a term frequency of 10. For the drug label datasets, a term frequency of 1 is selected to ensure no terms are excluded that could contribute to the prediction. However, for the CAMDA dataset, a term frequency of 10 is selected to save processing time as the CAMDA dataset is much larger with about 18 times more sample data.

Since both FDA and EMA datasets have the optimized model with the default condition with 10 iterations, using terms and phrases from FDA dataset to estimate the DILI prediction probability for each drug in the EMA dataset has 6% more inconsistency of DILI indicator in comparison with using terms and phrases from EMA dataset, with 84% consistency.

There are two types of classification errors in the model development: mistaking a drug that has DILI potential into non-DILI, and vice versa. From a clinical perspective, misclassifying a drug with DILI potential as non-DILI is a potentially more costly error as it could result in greater harm to patients. However, while misclassifying a non-DILI drug as having DILI potential group would likely cause less harm to patients, it would likely prove more costly for the pharmaceutical company. Like the concept of applicability domain (Tong et al., 2004), a Profit Matrix can be used to evaluate prediction performance by assuming different misclassification costs for DILI and non-DILI. The values in a Profit Matrix can be adjusted before or after a model is established to increase confidence in the classification in either direction, as shown in Supplementary Figure S4. When the probability threshold is changed from 0.5 to 0.75, the misclassification rate of DILI to non-DILI changes from 62 to 94, and the misclassification of non-DILI to DILI changes from 24 to 15which means more cases are classified to non-DILI cases. When the probability threshold is changed from 0.5 to 0.25, the misclassification rate of DILI to non-DILI cases decreases from 62 to 40, and the misclassification of non-DILI to DILI cases increases from 24 to 49, which means more cases are classified to DILI cases.

Comparing multiple predictive models showed that XGBoost had the best validated AUC, followed closely by boosted trees. XGBoost and boosted trees are powerful ensemble methods that combine multiple decision trees to create accurate predictive models. Bootstrap is a resampling technique used for model validation and uncertainty estimation. Decision trees are intuitive and interpretable but may suffer from overfitting. Neural networks offer the ability to model complex relationships but require large amounts of data and computational resources. SVMs provide robust classification but can be computationally expensive. The choice of predictive model depends on the specific problem, available data, interpretability requirements, and computational resources.

For drug labels we used a basic text mining bag-of-words approach. This method evaluates the content of the document and is blind to ordering, conditional statements, or sentiment. The language used for drug label warning and precautions is straightforward, making these documents ideal for this type of analysis. In contrast, publication abstracts are more complex and written by the authors with varying language skills and cultural backgrounds. Notably, our model is robust and demonstrates similar performance on both the CAMDA dataset and the drug labeling datasets. Other text mining methods that can handle more complicated grammar or negative sentences in natural language processing, such as the transformer neural networks of general domain BERT or specialized BERT (Shi et al., 2023; ValizadehAslani et al., 2023). The deep learning with Python codes such as BERT, require programming skills and a substantial amount of computing resources. The XGBoost can run much faster (a few minutes) using the document term matrix that were generated by Text Explorer in JMP Pro to predict DILI, while BERT needs much longer time (hours). While an optimal prediction strategy is likely to be an ensemble neural net and boosted trees (Shwartz-Ziv and Armon, 2022), we did not explore this in the present study. We also acknowledge potential improvement in performance for drug labelling can be obtained using transformer BERT-style language models we are pursuing this in additional research.

Here, we developed an automatic text classification of DILI using DTM and XGBoost, and it was successfully applied to analyze drug labels from FDA and EMA and literature abstracts from CAMDA. The XGBoost with text-based tabular features approach demonstrated here can be applied to other text processing and classification tasks and offers a non-code solution for scientists and researchers to access AI and ML technologies for natural language processing.

## Data availability statement

The original contributions presented in the study are included in the article/Supplementary material, further inquiries can be directed to the corresponding author.

## Author contributions

MC: Writing – original draft, Writing – review & editing. YW: Writing – original draft, Writing – review & editing. BW: Writing – original draft, Writing – review & editing. ZL: Writing – original draft, Writing – review & editing. JX: Writing – original draft, Writing – review & editing. ST: Writing – original draft, Writing – review & editing. TP: Writing – original draft, Writing – review & editing. TD: Writing – original draft, Writing – review & editing. NM: Writing – original draft, Writing – review & editing. WT: Writing – original draft, Writing – review & editing. RW: Writing – original draft, Writing – review & editing. WB: Writing – original draft, Writing – review & editing.

## Footnotes

^1^ https://nctr-crs.fda.gov/fdalabel/ui/search

^2^ https://www.medicines.org.uk

^3^ http://www.camda.info/

^4^ https://pubmed.ncbi.nlm.nih.gov/
