# Supplementary figures and images for: Automatic text classification of drug-induced liver injury using document-term matrix and XGBoost
Source: Front Artif Intell. 2024 Jun 3;7:1401810. doi: 10.3389/frai.2024.1401810 (PMC11181907; doi:10.3389/frai.2024.1401810)

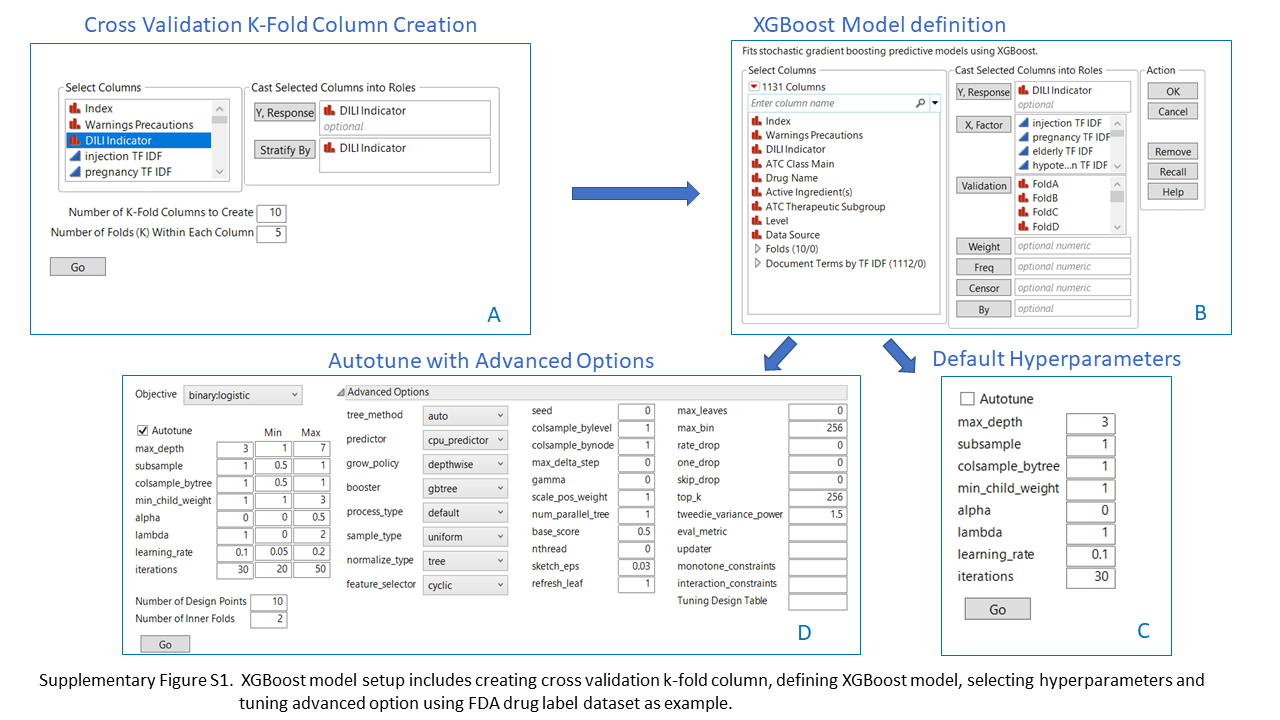

Supplement: Supplementary file 1 [file Image_1.tif]

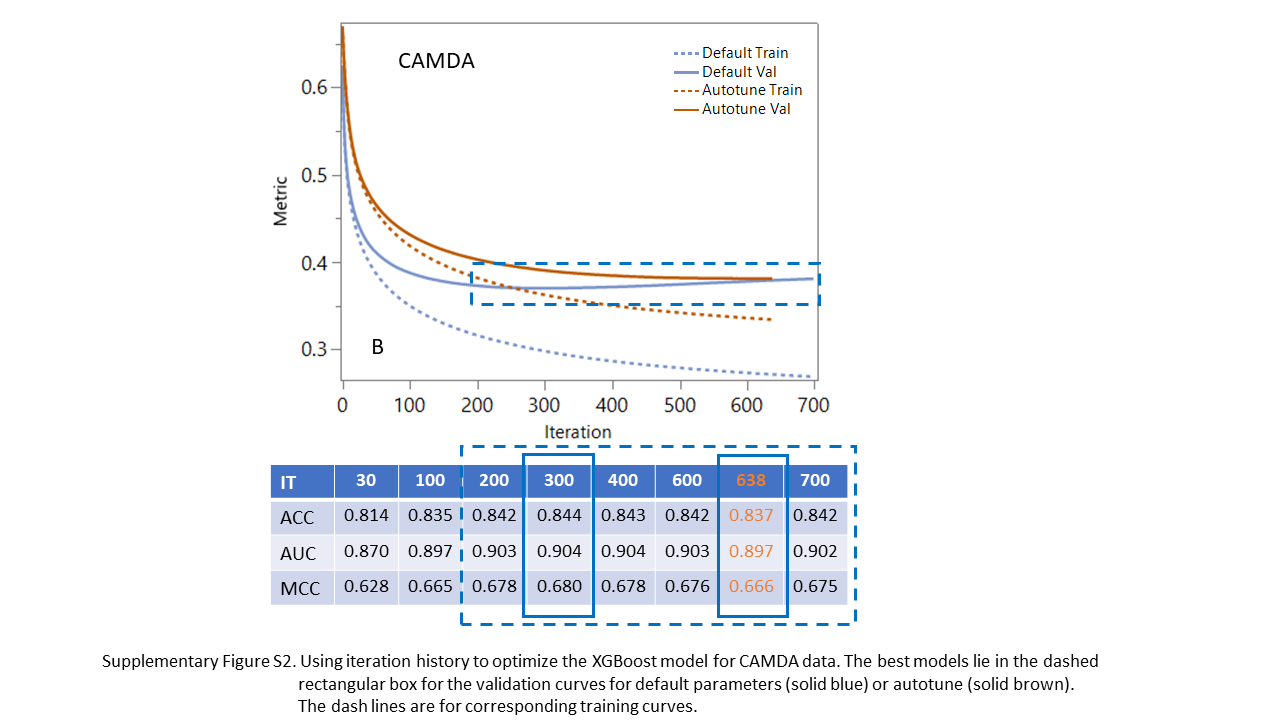

Supplement: Supplementary file 2 [file Image_2.tif]

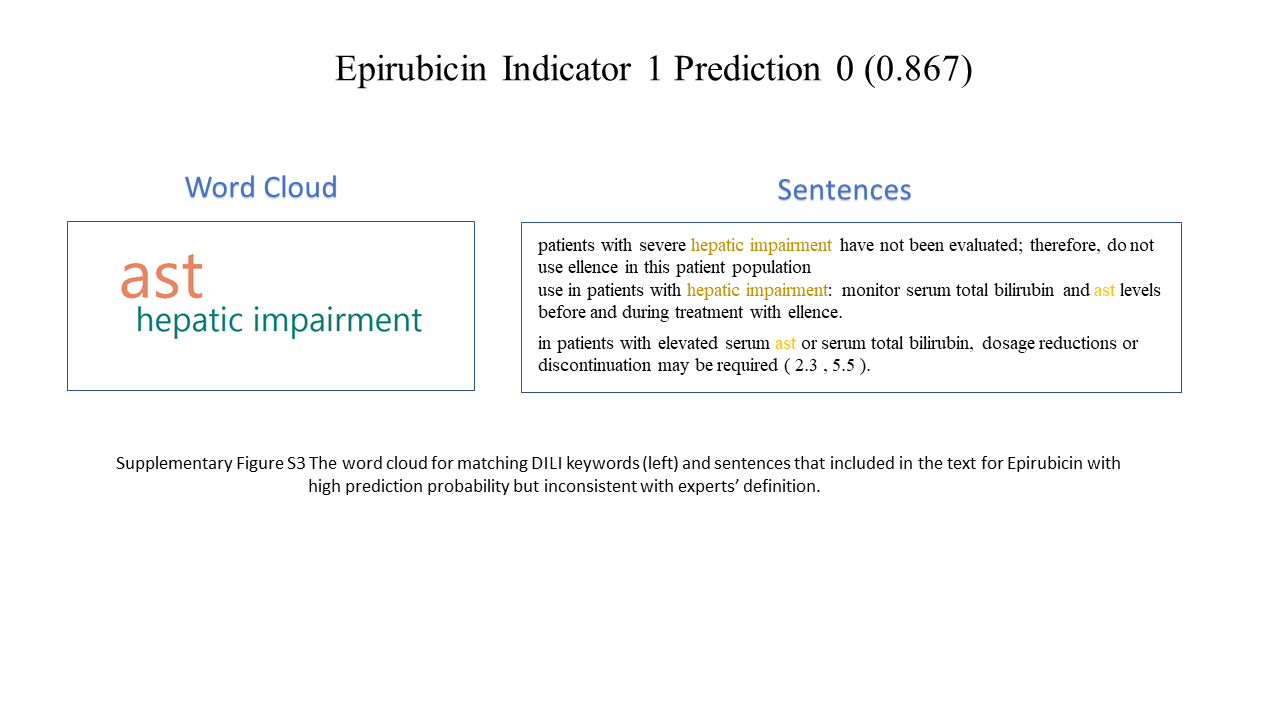

Supplement: Supplementary file 3 [file Image_3.tif]

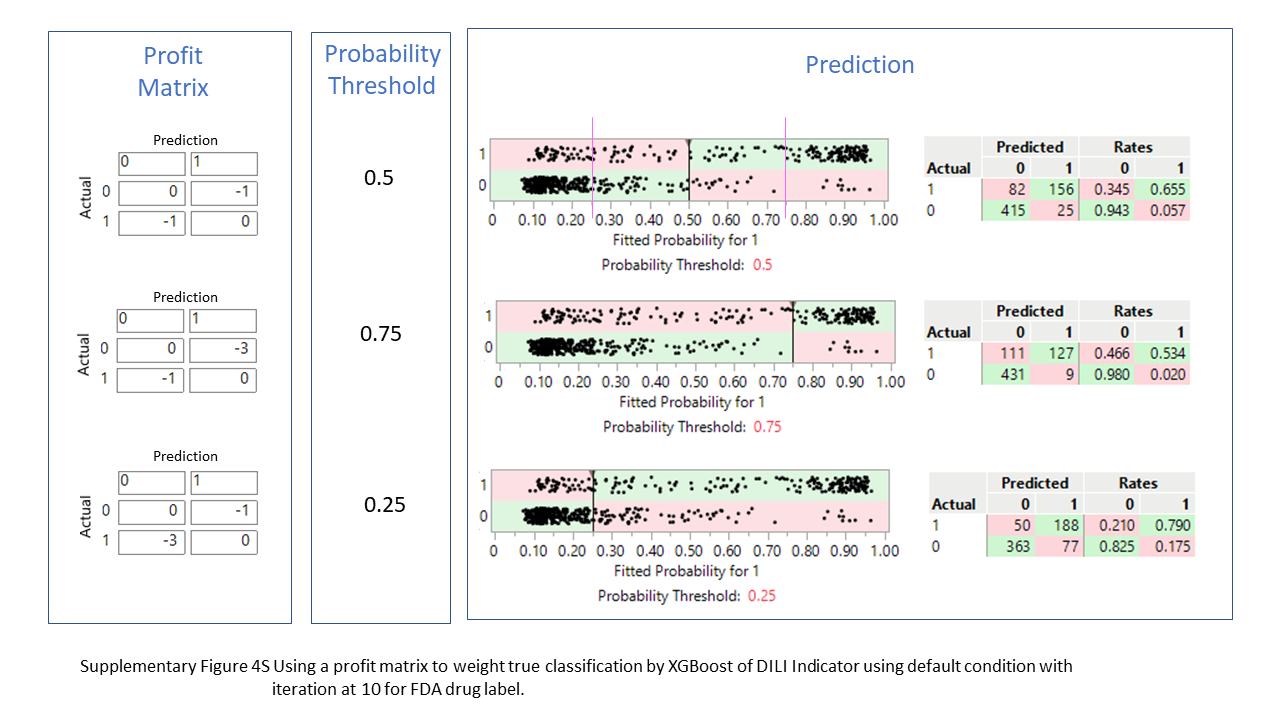

Supplement: Supplementary file 4 [file Image_4.tif]
