# Supplementary material for: Automatic text classification of drug-induced liver injury using document-term matrix and XGBoost
Source: Front Artif Intell. 2024 Jun 3;7:1401810. doi: 10.3389/frai.2024.1401810 (PMC11181907; doi:10.3389/frai.2024.1401810)
Supplement: Supplementary file 5 [file Table_1.pdf]

Supplementary Table S1. XGBoost hyperparameters for FDA, EMA and CAMDA datasets.

Dataset	Parameters	Max_ depth	Subsample	Colsample_ bytree	Min_child_ weight	Alpha	Lambda	Learning_ rate	iterations
FDA Drug Label	Default	6	1	1	1	0	1	0.3	10
	Autotune	6	0.791	0.730	1.474	0.412	0.420	0.170	35
EMA Drug Label	Default	6	1	1	1	0	1	0.3	10
	Autotune	6	0.831	0.570	1.358	0.301	0.364	0.195	36
CAMDA	Default	6	1	1	1	0	1	0.3	300
	Autotune	7	0.588	0.946	1.203	0.015	1.902	0.088	638
